# A Jeffrey Fluid Model for a Porous-walled Channel: Application to Flat Plate Dialyzer

**DOI:** 10.1038/s41598-019-52346-8

**Published:** 2019-11-04

**Authors:** M. Kahshan, D. Lu, A. M. Siddiqui

**Affiliations:** 1 Faculty of Science, Jiangsu University, Zhenjiang, Jiangsu 212013 P.R. China; 2 Department of Mathematics, COMSATS University Islamabad, Abbottabad Campus, Abbottabad, 22060 Pakistan; 3 Pennsylvania State University, York Campus, Edgecomb, 1703 USA

**Keywords:** Applied mathematics, Fluid dynamics

## Abstract

Creeping motion of a Jeffrey fluid in a small width porous-walled channel is presented with an application to flow in flat plate hemodialyzer. Darcy’s law is used to characterize the fluid leakage through channel walls. Using suitable physical approximations, approximate analytical solution of equations of motion is obtained by employing perturbation method. Expressions for velocity field and the hydrostatic pressure are obtained. Effects of filtration coefficient, the inlet pressure and Jeffrey fluid parameters on the flow characteristics are discussed graphically. The derived results are used to study the flow of filtrate in a flat plat hemodialyzer. Using the derived solutions, theoretical values of the filtration rate and the mean pressure difference in the hemodialyzer are calculated. On comparing the computed results with the available experimental data, a reasonable agreement between the two is found. It is concluded that the presented model can be used to study the hydrodynamical aspects of the fluid flow in a flat plate hemodialyzer.

## Introduction

The study of hydrodynamics of fluid flow in porous-walled channels and tubes have been remained a subject of interest for researchers since decades. This is because of the occurrence of such flows in many areas of science and engineering, particularly in those processes where mass transfer and filtration are encountered. Desalination process due to reverse osmosis, transpiration cooling, ultrafiltration in the tubules of glomerulus and fluid reabsorption through permeable walls of renal proximal tubule in kidneys, and blood filtration during hemodialysis in an artificial kidney are examples of such flows^1–6^. A common characteristic of such flows is the direct inapplicability of the usual Poiseuille law^7^ in these situations because of the existence of non-zero velocity components of fluid in both axial and normal directions. This happens because of the fluid leakage through porous walls of the channel.

Literature survey reveals that various researchers have attempted the problem of fluid flow in porous-walled channels and tubes^5,8–12^ by assuming different forms of fluid reabsorption at the walls. Berman^8^, Sellars^9^, and Yuan^10^ have studied the laminar flow of incompressible Newtonian fluid in a uniformly porous-walled channel. Solutions were obtained for a uniform suction or injection case using the regular perturbation method. Kozinski *et al*.^11^ and Siddiqui *et al*.^12^ also studied the creeping flow of a viscous fluid in a permeable channel. Exact solutions were found for the case in which the leakage has exponential and linear decaying rate along the channel length. Flow of a viscous fluid in a porous-walled channel was also studied by Marshall *et al*.^5^ who assumed that the fluid leakage across the channel walls is proportional to the pressure differences across the walls. For further studies of fluid flows under different flow conditions authors would refer the reader to^13–19^.

In the literature presented above, all studies were performed for the Newtonian fluid model only. Despite the fact that most of the industrial and biological fluids are non-Newtonian^20^ and the classical Newton’s law of viscosity fails to describe the complex rheological properties of non-Newtonian fluids, a very little work has been done in order to study the non-Newtonian fluids flow in porous-walled channels and tubes. Amongst many non-Newtonian fluid models, one is the Jeffrey fluid model that has attracted many researchers due to its consideration as a better model for physiological fluids^21–23^. The Jeffrey model is recognized as a generalization of the frequently used Newtonian fluid model because of the fact tat its constitutive equation can be reduced to that of the Newtonian model’s as a special case. The Jeffrey fluid model is capable of describing the stress relaxation property of non-Newtonian fluids, which the usual viscous fluid model cannot describe. Class of non-Newtonian fluids having the characteristic memory time scale, also known as the relaxation time, can be described well by the Jeffrey fluid model. Therefore, this article is devoted to the study of an incompressible Jeffrey fluids’ flow in a porous-walled channel of small width. Fluid absorption at the channel wall is taken in accordance with the Darcy’s law. The approach of the present analysis seems better from that of ^2,3,11,24^. In the present approach, the fluid loss at the wall is taken as function of the wall permeability, the flow rate then determined can be found to decay linearly or exponentially along the channel wall as special case in our study. Whereas, in^2,3,11,24^ a flow rate that decays linearly or exponentially, along the channel and tube was assumed in advance.

## Problem Formulation

**Figure 1:**
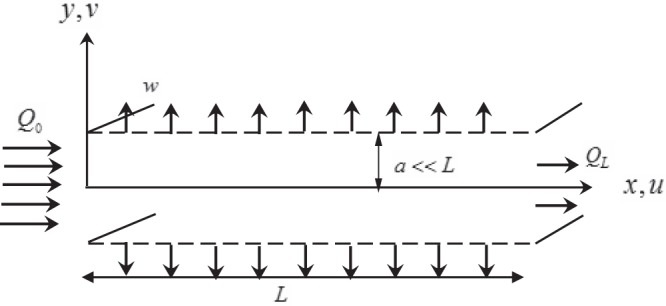
Geometry of the flow.

Let us consider the steady and creeping motion of an incompressible Jeffrey fluid in a porous-walled channel comprising of two equidistant parallel flat plates of length *L* distance 2*a* apart. A Cartesian coordinate system $(\tilde{x},\tilde{y})$ is chosen in a such that the axis of symmetry lies at *y* = 0 as shown in Fig. 1. It is assumed that the fluid moves only due to the hydrostatic pressure. Hydrostatic and osmotic pressures outside the channel are also assumed ot be constant. The flow is governed by the following equations

1 $$\frac{\partial \tilde{p}}{\partial \tilde{x}}=\frac{\partial }{\partial \tilde{x}}({\tilde{S}}_{\tilde{x}\tilde{x}})+\frac{\partial }{\partial \tilde{y}}({\tilde{S}}_{\tilde{x}\tilde{y}}),$$

2 $$\frac{\partial \tilde{p}}{\partial \tilde{y}}=\frac{\partial }{\partial \tilde{x}}({\tilde{S}}_{\tilde{y}\tilde{x}})+\frac{\partial }{\partial \tilde{y}}({\tilde{S}}_{\tilde{y}\tilde{y}}),$$

3 $$\frac{\partial \tilde{u}}{\partial \tilde{x}}+\frac{\partial \tilde{v}}{\partial \tilde{y}}=0,$$

where $\tilde{p}$ is the hydrostatic pressure of the fluid, ${\tilde{S}}_{ij},i,j=\tilde{x},\tilde{y}$ are components of the extra stress tensor, $\tilde{u}$ and $\tilde{v}$ are components of the velocity vector $\tilde{{\bf{V}}}(\tilde{x},\tilde{y})$ in the axial and transverse directions, respectively.

The extra stress tensor $\tilde{S}$ for the Jeffrey fluid is given by the following equation^21–23^

4 $$\tilde{{\bf{S}}}=\frac{\mu }{1+{\lambda }_{1}}[\dot{{\bf{A}}}+{\tilde{\lambda }}_{2}\ddot{{\bf{A}}}],$$

where *μ* is the dynamic viscosity of the fluid, $\dot{{\bf{A}}}$ is the shear rate, ${\tilde{\lambda }}_{2}$ is the retardation time*, λ*_1_ is the ratio of relaxation time to the retardation time, and $\ddot{{\bf{A}}}$ represents differentiation of $\dot{{\bf{A}}}$. The initial and boundary conditions corresponding to the prescribed flow are

5 $$\tilde{v}(\tilde{x},0)=0,$$

6 $$\partial \tilde{u}(\tilde{x},0)/\partial \tilde{y}=0,$$

7 $$\tilde{v}(\tilde{x},a)=\frac{{L}_{p}}{\mu t}[\tilde{p}(\tilde{x},a)-{p}_{m}],$$

8 $$\tilde{u}(\tilde{x},a)=0,$$

9 $$\frac{1}{a}{\int }_{0}^{a}\,\tilde{p}(0,\tilde{y})d\tilde{y}={\tilde{p}}_{i},$$

10 $$2w{\int }_{0}^{a}\,\tilde{u}(0,\tilde{y})d\tilde{y}={\tilde{Q}}_{i}.$$

Equations (5) and (6) are the symmetry conditions at the center line of the channel. Equation (7) is the consequence of the Darcy’s law at the permeable wall of the channel, where *L*_*p*_ is the mechanical filtration coefficient of the channel wall which is usually measured in the units of *cm*^2^(*L*_*p*_/*μt* is called the hydraulic permeability of the channel wall), *t* is the wall thickness, and *p*_*m*_ can be visualized as the back pressure that opposes the fluid leakage and it is equal to the difference of hydrostatic and the osmotic pressures outside the channel wall. Equation (8) is the no slip condition at the wall, whereas Equations (9 and 10) are the inlet conditions. In these equations ${\tilde{p}}_{i}$ is the mean pressure and ${\tilde{Q}}_{i}$ is the flow rate at the inlet of the channel at $\tilde{x}=0$.

## Dimensionless Formulation

Following parameters are used to transform equations into dimensionless form:

11 $$x=\frac{\tilde{x}}{L};\,y=\frac{\tilde{y}}{a};\,u(x,y)=\frac{{a}^{2}\tilde{u}}{{\tilde{Q}}_{i}};\,v(x,y)=\frac{aL\tilde{v}}{{\tilde{Q}}_{i}}.$$

Using the above quantities, Eqs (1−3) take the following form

12 $$\frac{\partial p}{\partial x}=\delta \frac{\partial }{\partial x}({S}_{xx})+\frac{\partial }{\partial y}({S}_{xy}),$$

13 $$\frac{\partial p}{\partial y}={\delta }^{2}\frac{\partial }{\partial x}({S}_{yx})+\delta \frac{\partial }{\partial y}({S}_{yy}),$$

14 $$\frac{\partial u}{\partial x}+\frac{\partial v}{\partial y}=0,$$

where $\delta =\frac{a}{L}$ is the ratio of channel height to its length, ${\bf{S}}=\frac{{a}^{3}\tilde{{\bf{S}}}}{\mu {\tilde{Q}}_{i}}$ is the dimensional stress tensor and $p(x,y)=\frac{{a}^{4}}{\mu L{\tilde{Q}}_{i}}[\tilde{p}(\tilde{x},\tilde{y})-{p}_{m}]$. The initial and boundary conditions Eqs (5−10) are transformed into the following dimensionless form

15 $$v(x,0)=0,$$

16 $$\partial u(x,0)/\partial x=0,$$

17 $$v(x,1)=Kp(x,1),$$

18 $$u(x,1)=0,$$

19 $${\int }_{0}^{1}p(0,y)dy={p}_{i},$$

20 $$2A{\int }_{0}^{1}\,u(0,y)dy=1,$$

where $K=\frac{{L}_{p}{L}^{2}}{{a}^{3}t}$ is the dimensionless wall filtration parameter and ${\lambda }_{2}=\frac{{\tilde{\lambda }}_{2}{\tilde{Q}}_{i}}{{a}^{2}L}$ is the Jeffrey fluid parameter in dimensionless form, $A=\frac{w}{a}$ is the ratio of channel width to height and ${Q}({x})=\frac{\tilde{{Q}}(\tilde{{x}})}{{\tilde{{Q}}}_{{i}}}$.

Using Eq. (4) and the dimensionless parameters, the components of the stress tensor **S** can be readily obtained in the following dimensionless form

21 $${S}_{xx}=\frac{2\delta }{1+{\lambda }_{1}}[1+{\lambda }_{2}(u\frac{\partial }{\partial x}+v\frac{\partial }{\partial y})](\frac{\partial u}{\partial x}),$$

22 $${S}_{xy}=\frac{1}{1+{\lambda }_{1}}[1+{\lambda }_{2}(u\frac{\partial }{\partial x}+v\frac{\partial }{\partial y})](\frac{\partial u}{\partial y}+{\delta }^{2}\frac{\partial v}{\partial x}),$$

23 $${S}_{yx}=\frac{1}{1+{\lambda }_{1}}[1+{\lambda }_{2}(u\frac{\partial }{\partial x}+v\frac{\partial }{\partial y})](\frac{\partial u}{\partial y}+{\delta }^{2}\frac{\partial v}{\partial x}),$$

24 $${S}_{yy}=\frac{2{\delta }^{2}}{1+{\lambda }_{1}}[1+{\lambda }_{2}(u\frac{\partial }{\partial x}+v\frac{\partial }{\partial y})](\frac{\partial v}{\partial y}).$$

## Solution to the Problem

The set of Eqs (12−14) contains nonlinear coupled partial differential equations in three unknowns *u*, *v* and *p* and it is not possible to determine an exact solution of it. Since the channel is narrow and has small width as compared with its length, therefore the dimensionless parameter *δ* is very small. Thus it seems reasonable to ignore terms of the order *δ*^2^ and higher order. Note that this is also true for the flat plate hemodialyzer, for which the parameter *δ*^2^ is of the order 10^−8^ (see the data given in Table 1 and ^25–27^).

**Table 1:** Experimental data for the RP kidney ^4,6,29^.

Description	Symbol	Numerical data
Membrane length	*L*	42 cm
Membrane thickness	*t*	2.59 × 10^−3^ cm
Membrane width	*w*	11.6 cm
Blood compartments		8
Height of the blood half channel	*a*	9 × 10^−3^ cm
Entrance trans-membrane pressure difference	${\bar{P}}_{0}-{P}_{T}$	150 mm Hg
Viscosity of the fluid	*μ*	6.9 × 10^−3^ dynes-sec/cm^2^
Entrance volume flow rate	8*Q*_0_	160 ml/min
Ultrafiltration rate	8*Q*_*w*_	200 ml/hr

Now expanding *u*, *v* and *p* in the power series of the dimensionless Jeffrey fluid parameter as

25 $$(u,v,p)=({u}_{0},{v}_{0},{p}_{0})+{\lambda }_{2}({u}_{1},{v}_{1},{p}_{1})+O({\lambda }_{2}^{2}),$$

we obtain the following systems of initial-boundary-value problem up-to the Order (*λ*_2_):

### Zeroth order system

 

26 $$\frac{\partial {p}_{0}}{\partial y}=0$$

27 $$\frac{\partial {p}_{0}}{\partial x}=\frac{1}{1+{\lambda }_{1}}\frac{{\partial }^{2}{u}_{0}}{\partial {y}^{2}},$$

28 $$\frac{\partial {u}_{0}}{\partial x}+\frac{\partial {v}_{0}}{\partial y}=0,$$

29 $${v}_{0}(x,0)=0,$$

30 $$\partial {u}_{0}(x,0)/\partial x=0,$$

31 $${v}_{0}(x,1)=K{p}_{0}(x,1),$$

32 $${u}_{0}(x,1)=0,$$

33 $${\int }_{0}^{1}\,{p}_{0}(0,y)dy={p}_{i},$$

34 $$2A{\int }_{0}^{1}\,{u}_{0}(0,y)dy=1.$$

### First order system

 

35 $$\frac{\partial {p}_{1}}{\partial y}=0$$

36 $$\frac{\partial {p}_{1}}{\partial x}=\frac{1}{1+{\lambda }_{1}}\frac{\partial }{\partial y}[\frac{\partial {u}_{1}}{\partial y}+({u}_{0}\frac{\partial }{\partial x}+{v}_{0}\frac{\partial }{\partial y})(\frac{\partial {u}_{0}}{\partial y})],$$

37 $$\frac{\partial {u}_{1}}{\partial x}+\frac{\partial {v}_{1}}{\partial y}=0,$$

38 $${v}_{1}(x,0)=0,$$

39 $$\partial {u}_{1}(x,0)/\partial x=0,$$

40 $${v}_{1}(x,1)=K{p}_{1}(x,1),$$

41 $${u}_{1}(x,1)=0,$$

42 $${\int }_{0}^{1}\,{p}_{1}(0,y)dy=0,$$

43 $${\int }_{0}^{1}\,{u}_{1}(0,y)dy=0.$$

### Zeroth order solution

The exact solution of the zeroth order system is given in the following

44 $${u}_{0}(x,y)=\frac{1+{\lambda }_{1}}{2}\frac{d{p}_{0}}{dx}({y}^{2}-1),$$

45 $${v}_{0}(x,y)=-\,\frac{1+{\lambda }_{1}}{6}\frac{{d}^{2}{p}_{0}}{d{x}^{2}}({y}^{3}-3y),$$

46 $${p}_{0}(x)=(\frac{{p}_{i}}{2}+\frac{\xi }{4AK}){e}^{-\xi x}+(\frac{{p}_{i}}{2}-\frac{\xi }{4AK}){e}^{\xi x},$$

where $$\xi =\sqrt{\frac{3K}{1+{\lambda }_{1}}}$$.

### First order solution

The exact solution of the first order system is given in the following

47 $${u}_{1}(x,y)=\frac{1+{\lambda }_{1}}{2}\frac{d{p}_{1}}{dx}({y}^{2}-1)-\frac{{(1+{\lambda }_{1})}^{2}}{12}\frac{d{p}_{0}}{dx}\frac{{d}^{2}{p}_{0}}{d{x}^{2}}({y}^{4}-1),$$

48 $$\begin{array}{rcl}{v}_{1}(x,y) & = & -\frac{1+{\lambda }_{1}}{6}\frac{{d}^{2}{p}_{1}}{d{x}^{2}}({y}^{3}-3y)\\  &  & +\frac{{(1+{\lambda }_{1})}^{2}}{60}\frac{d}{dx}(\frac{d{p}_{0}}{dx}\frac{{d}^{2}{p}_{0}}{d{x}^{2}})({y}^{5}-5y),\end{array}$$

49 $${p}_{1}(x)={C}_{3}{e}^{-\xi x}+{C}_{4}{e}^{\xi x}+\frac{2}{15}{\xi }^{2}(1+{\lambda }_{1})({C}_{1}^{2}{e}^{-2\xi x}+{C}_{2}^{2}{e}^{2\xi x}),$$

where the constants *C*_1_, *C*_2_, *C*_3_ and *C*_4_ are respectively given by the following expressions

50 $${C}_{1}=\frac{{p}_{i}}{2}+\frac{\xi }{4AK},$$

51 $${C}_{2}=\frac{{p}_{i}}{2}-\frac{\xi }{4AK},$$

52 $${C}_{3}={\xi }^{2}(1+{\lambda }_{1})[\frac{pi\xi }{20AK}+\frac{1}{15}(3{C}_{1}^{2}-{C}_{2}^{2})],$$

53 $${C}_{4}={\xi }^{2}(1+{\lambda }_{1})[-\frac{pi\xi }{20AK}+\frac{1}{15}({C}_{1}^{2}-3{C}_{2}^{2})].$$

Summing up, the approximate solution upto the order *λ*_2_ can be readily obtained as

54 $$\begin{array}{rcl}u(x,y) & = & {u}_{0}(x,y)+{\lambda }_{2}{u}_{1}(x,y),\\  & = & \frac{1}{2}(1+{\lambda }_{1})\frac{dp}{dx}({y}^{2}-1)\\  &  & -\frac{1}{12}{(1+{\lambda }_{1})}^{2}{\lambda }_{2}\frac{d{p}_{0}}{dx}\frac{{d}^{2}{p}_{0}}{d{x}^{2}}({y}^{4}-1).\end{array}$$

55 $$\begin{array}{rcl}v(x,y) & = & {v}_{0}(x,y)+{\lambda }_{2}{v}_{1}(x,y),\\  & = & -\frac{1}{6}(1+{\lambda }_{1})\frac{{d}^{2}p}{d{x}^{2}}({y}^{3}-3y)\end{array}$$

56 $$+\frac{1}{60}{(1+{\lambda }_{1})}^{2}{\lambda }_{2}\frac{d}{dx}(\frac{d{p}_{0}}{dx}\frac{{d}^{2}{p}_{0}}{d{x}^{2}})({y}^{5}-5y).$$

57 $$\begin{array}{rcl}p(x) & = & {p}_{0}(x)+{\lambda }_{2}{p}_{1}(x),\\  & = & ({C}_{1}+{\lambda }_{2}{C}_{3}){e}^{-\xi x}+({C}_{2}+{\lambda }_{2}{C}_{4}){e}^{\xi x}\\  &  & +\frac{2}{15}{\xi }^{2}(1+{\lambda }_{1}){\lambda }_{2}({C}_{1}^{2}{e}^{-2\xi x}+{C}_{2}^{2}{e}^{2\xi x}).\end{array}$$

It is remarkable to point out that in the above velocity and pressure profiles, we get contribution of both parameters *λ*_1_ and *λ*_2_ of the Jeffrey fluid. For the limiting case when *λ*_1_ → 0 and *λ*_2_ → 0, the presented solutions reduce to the solution for Newtonian fluid flow in a permeable channel.

## Expressions for Various Quantities of Interest

The dimensionless mean pressure $\bar{p}(x)$ taken over any cross-section of the channel is defined as

$$\begin{array}{rcl}\bar{p}(x) & = & \frac{1}{2}{\int }_{-1}^{1}\,p(x,y)dy\\  & = & p(x)\end{array}$$

Hence the difference of mean pressure Δ*p*(*x*) can be obtained as

58 $$\begin{array}{rcl}\Delta p(x) & = & \bar{p}(0)-\bar{p}(x),\\  & = & {p}_{i}-({C}_{1}+{\lambda }_{2}{C}_{3}){e}^{-\xi x}-({C}_{2}+{\lambda }_{2}{C}_{4}){e}^{\xi x}\\  &  & -\frac{2}{15}{\xi }^{2}(1+{\lambda }_{1}){\lambda }_{2}({C}_{1}^{2}{e}^{-2\xi x}+{C}_{2}^{2}{e}^{2\xi x}).\end{array}$$

The non-dimensional wall shear stress may be obtained as

59 $$\begin{array}{rcl}{\tau }_{w}(x) & = & -{S}_{yx}{|}_{y=1},\\  & = & -\frac{1}{(1+{\lambda }_{1})}{[\frac{\partial u}{\partial y}+{\lambda }_{2}({u}_{0}\frac{{\partial }^{2}{u}_{0}}{\partial x\partial y}+{v}_{0}\frac{{\partial }^{2}{u}_{0}}{\partial {y}^{2}})]}_{y=1}.\end{array}$$

The dimensionless volume flow rate at any cross-section of the channel can be computed as

60 $$\begin{array}{rcl}Q(x) & = & A{\int }_{-1}^{1}\,u(x,y)dy\\  & = & -\frac{2}{3}A(1+{\lambda }_{1})[\frac{dp}{dx}-\frac{1}{5}{\lambda }_{2}(1+{\lambda }_{1})\frac{d{p}_{0}}{dx}\frac{{d}^{2}{p}_{0}}{d{x}^{2}}].\end{array}$$

The expression for leakage flux *q*(*x*) can be readily obtained as following

61 $$\begin{array}{rcl}q(x) & = & -\frac{dQ}{dx},\\  & = & \frac{2}{3}A(1+{\lambda }_{1})[\frac{{d}^{2}p}{d{x}^{2}}-\frac{1}{10}{\lambda }_{2}(1+{\lambda }_{1})\frac{{d}^{2}}{d{x}^{2}}{(\frac{d{p}_{0}}{dx})}^{2}].\end{array}$$

The fractional re-absorption (FR) is the amount of fluid that has been reabsorbed through the channel walls. It can be computed using the following expression

62 $$FR=\frac{Q(0)-Q(1)}{Q(0)}.$$

The streamlines can be determined by using the following relation

63 $$\frac{dx}{u}=\frac{dy}{v}.$$

Thus streamlines can be obtained up to the order *λ*_2_ by the following equation

64 $$10\frac{dp}{dx}({y}^{3}-3y)-{\lambda }_{2}(1+{\lambda }_{1})\frac{d{p}_{0}}{dx}\frac{{d}^{2}{p}_{0}}{d{x}^{2}}({y}^{5}-5y)={C}_{5},$$

where *C*_5_ is an arbitrary constant.

## Results and Discussion

This section describes effects of the inlet pressure *p*_*i*_, wall permeability parameter *K* and the Jeffrey fluid parameters *λ*_1_ and *λ*_2_ on the axial and normal velocity components *u*(*x*, *y*), *v*(*x*, *y*), the mean pressure difference Δ*p*(*x*) and the streamline patterns.

**Figure 2:**
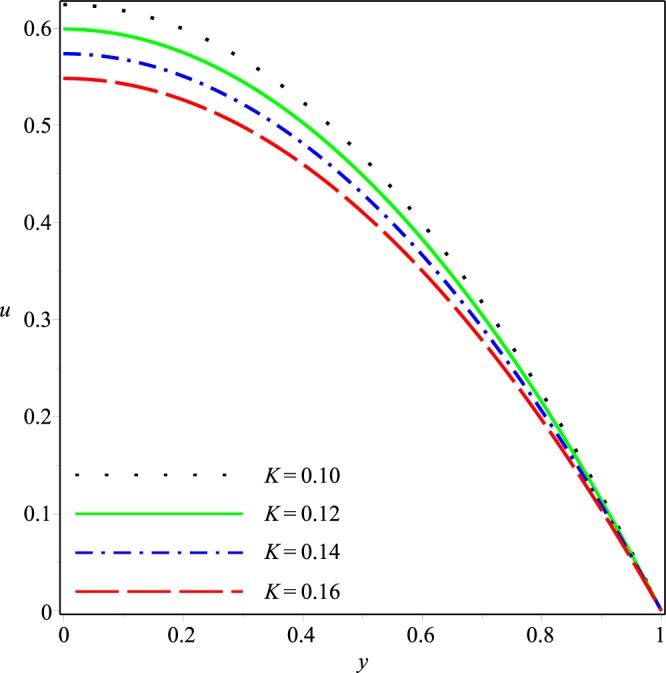
Effect of *K* on the axial velocity for *p*_*i*_ = 3, *λ*_1_ = 0.1, *λ*_2_ = 0.03.

**Figure 3:**
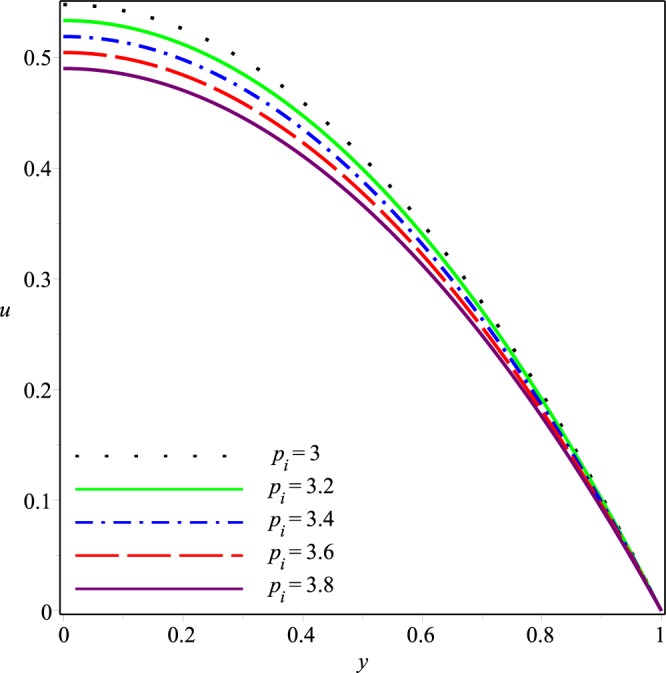
Effect of *p*_*i*_ on the axial velocity for *K* = 0.16, *λ*_1_ = 0.1, *λ*_2_ = 0.03.

**Figure 4:**
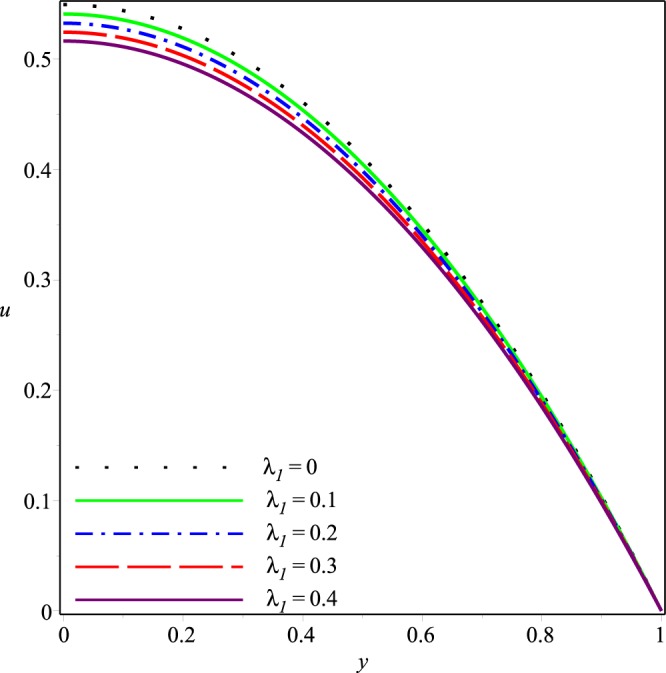
Effect of *λ*1 on the axial velocity for *p*_*i*_ = 3, *K* = 0.16, *λ*_2_ = 0.03.

**Figure 5:**
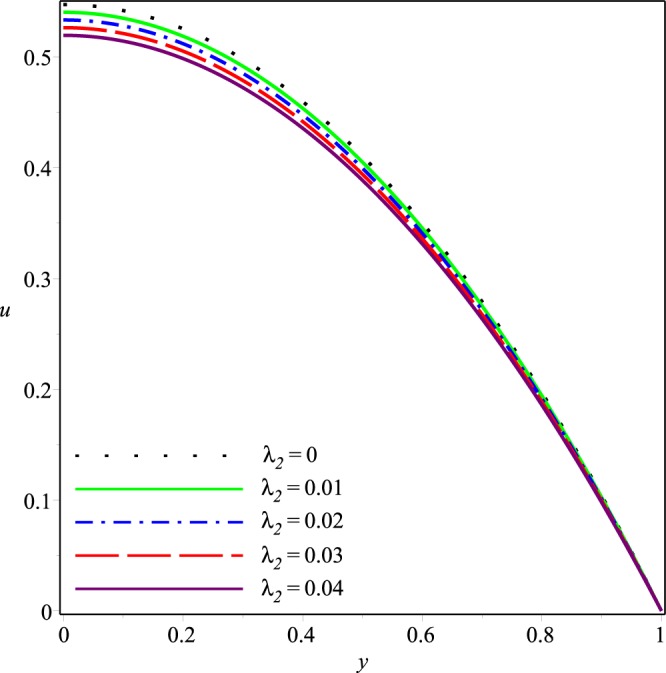
Effect of *λ*_2_ on the axial velocity for *p*_*i*_ = 3, *K* = 0.16, *λ*_1_ = 0.1.

Figures 2−5 present the variation of *u*(*x*, *y*) with *y* at the cross-section *x* = 0.3 of the channel for different values of *K*, *p*_*i*_, *λ*_1_ and *λ*_2_, respectively. A parabolic axial velocity is formed that has the maximum value at the center line of channel and is zero at the boundary. These figures reveal that the magnitude of axial velocity decreases rapidly as the inlet pressure and the wall permeability are increased. Similar effects are also noticed when the magnitudes of Jeffery fluid parameters is increased. However relatively small variations happen due to increase of *λ*_1_ and *λ*_2_ as compared to those due to *p*_*i*_ and *K*.

**Figure 6:**
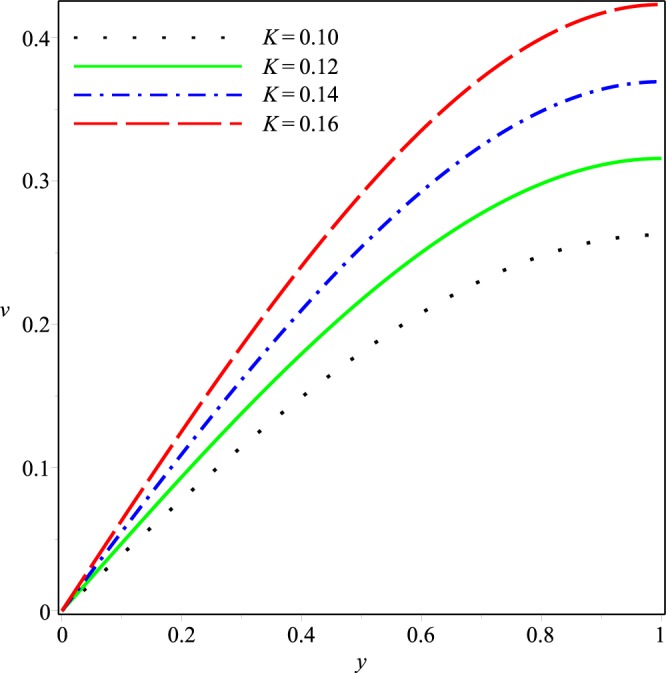
Effect of *K* on the normal velocity for *p*_*i*_ = 3, *λ*_1_ = 0.1, *λ*_2_ = 0.03.

**Figure 7:**
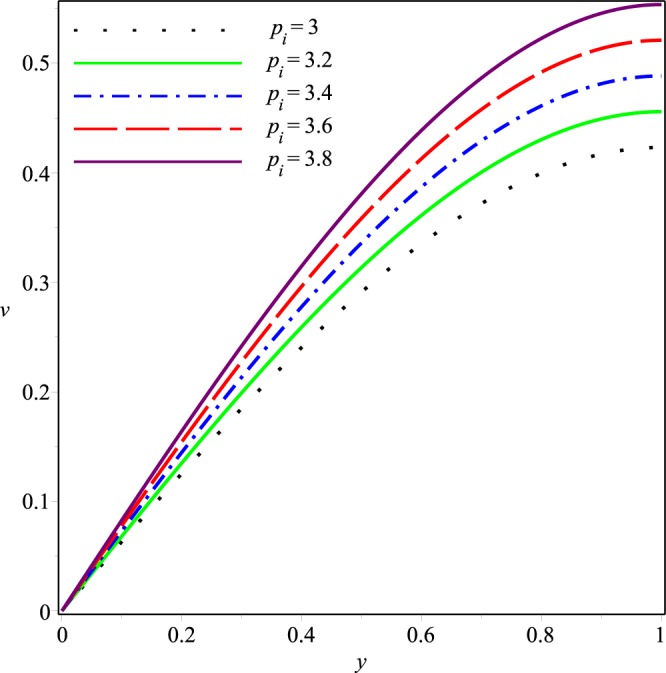
Effect of *p*_*i*_ on the normal velocity for *K* = 0.16, *λ*_1_ = 0.1, *λ*_2_ = 0.03.

**Figure 8:**
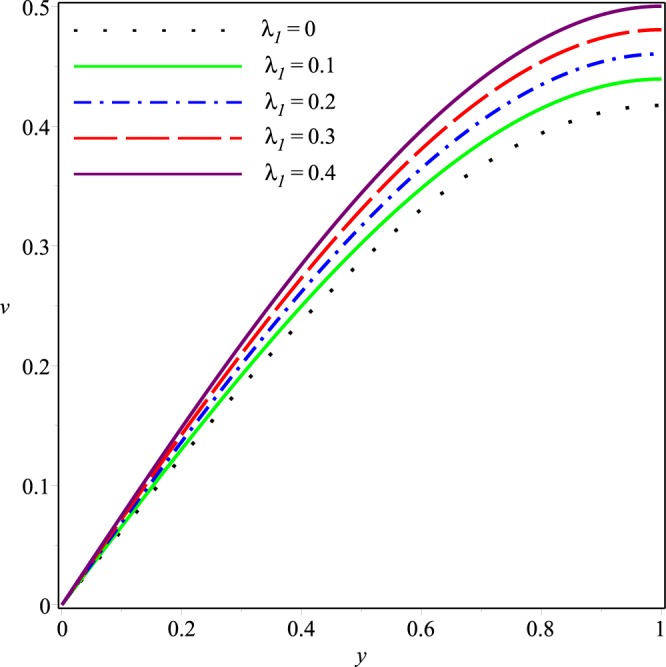
Effect of *λ*_1_ on the normal velocity for *p*_*i*_ = 3, *K* = 0.16, *λ*_2_ = 0.03.

**Figure 9:**
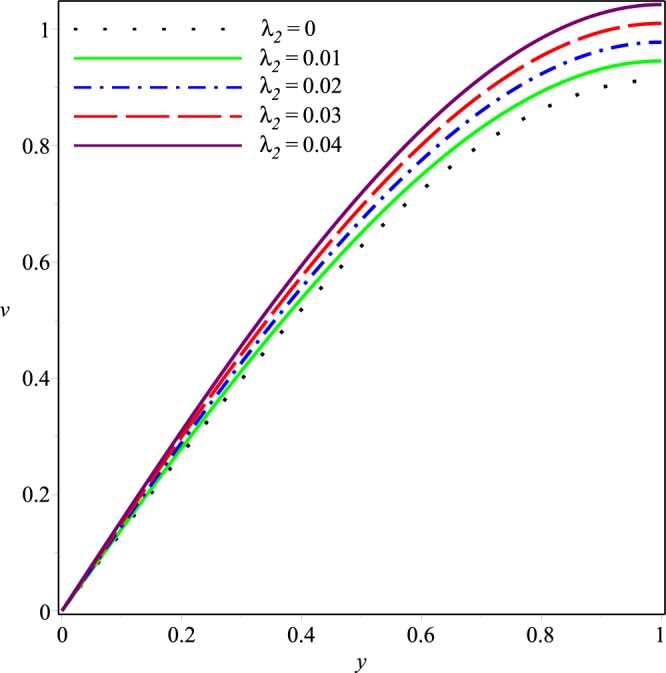
Effect of *λ*_2_ on the normal velocity for *p*_*i*_ = 3, *K* = 0.16, *λ*_1_ = 0.1.

Figures 6−9 present the variation of *v*(*x*, *y*) with *y* at the cross-section *x* = 0.3 of the channel for different values of *K*, *p*_*i*_, *λ*_1_ and *λ*_2_, respectively. Comparing with the axial velocity distribution, opposite effects of these parameters are observed on the normal velocity component. It can be seen clearly that *v* increases rapidly as the magnitudes of *p*_*i*_, *K*, *λ*_1_ and *λ*_2_ are increased. Thus, the wall seepage enhances by increasing these parameters. Effect of *λ*_1_ and *λ*_2_ on *v* are prominent as compared to those on *u*.

**Figure 10:**
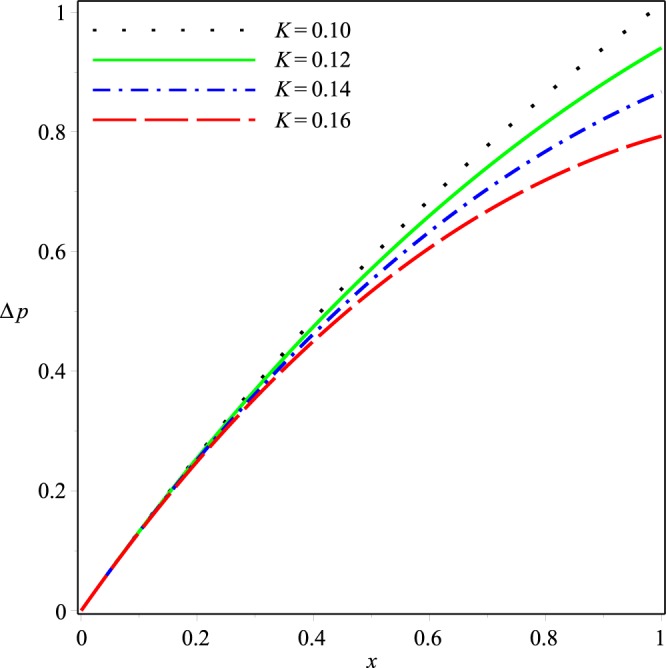
Effect of *K* on the mean pressure drop for *p*_*i*_ = 3, *λ*_1_ = 0.1, *λ*_2_ = 0.03.

**Figure 11:**
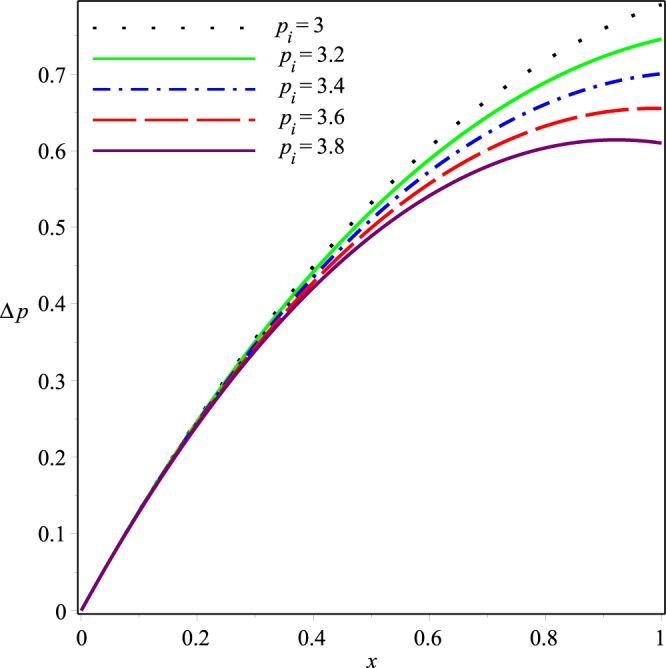
Effect of *p*_*i*_ on the mean pressure drop for *K* = 0.16, *λ*_1_ = 0.1, *λ*_2_ = 0.03.

**Figure 12:**
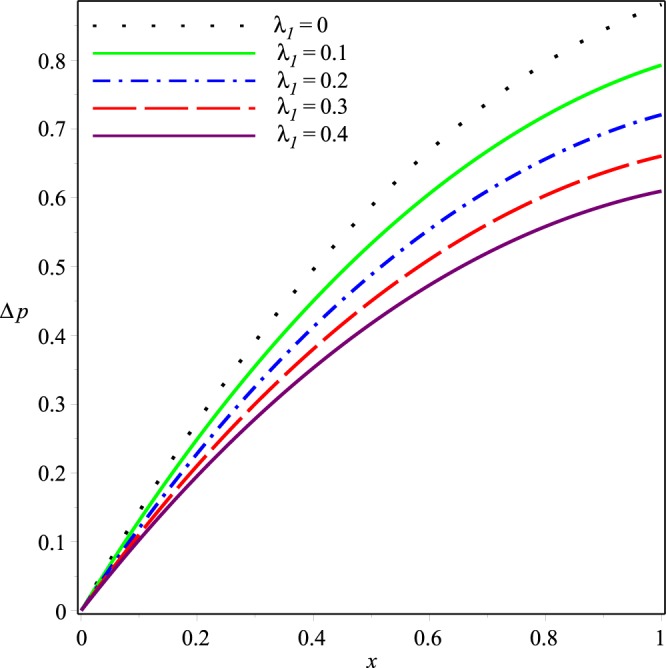
Effect of *λ*_1_ on the mean pressure drop for *p*_*i*_ = 3, *K* = 0.16, *λ*_2_ = 0.03.

**Figure 13:**
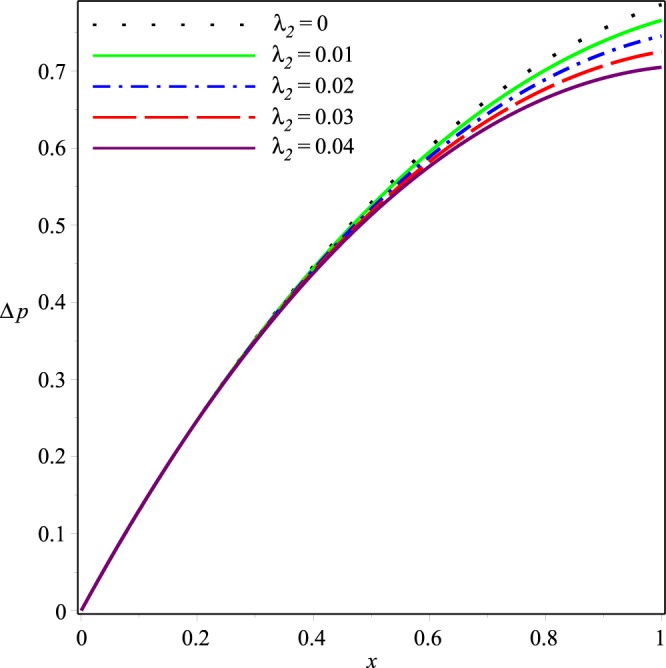
Effect of *λ*_2_ on the mean pressure drop for *p*_*i*_ = 3, *K* = 0.16, *λ*_1_ = 0.1.

Variations of the mean pressure difference in the axial direction for different values of *p*_*i*_, *K*, *λ*_1_ and *λ*_2_ are shown in Figs 10−13. These figures depict that Δ*p* decreases rapidly as the values of these parameters are increased. It can also be seen that variations in Δ*p* are dominant after the middle of the channel.

**Figure 14:**
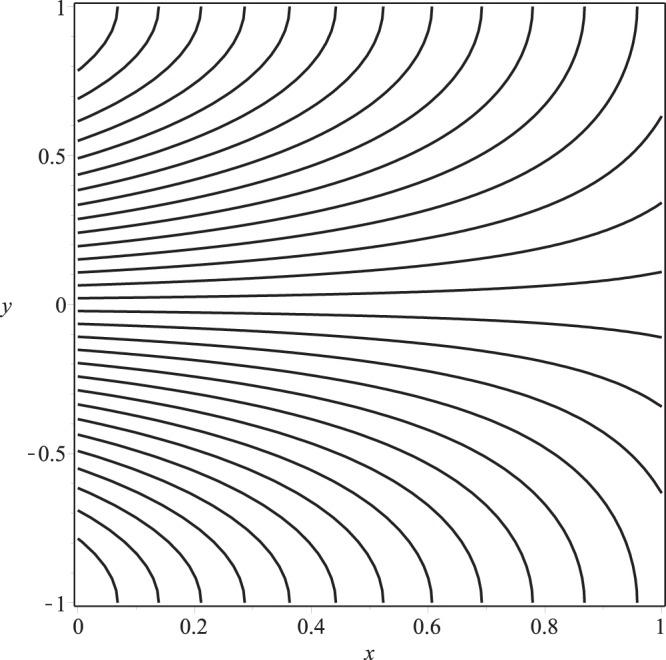
Streamline pattern of the flow or *p*_*i*_ = 3, K = 0.16, *λ*_1_ = 0.1, *λ*_2_ = 0.03.

**Figure 15:**
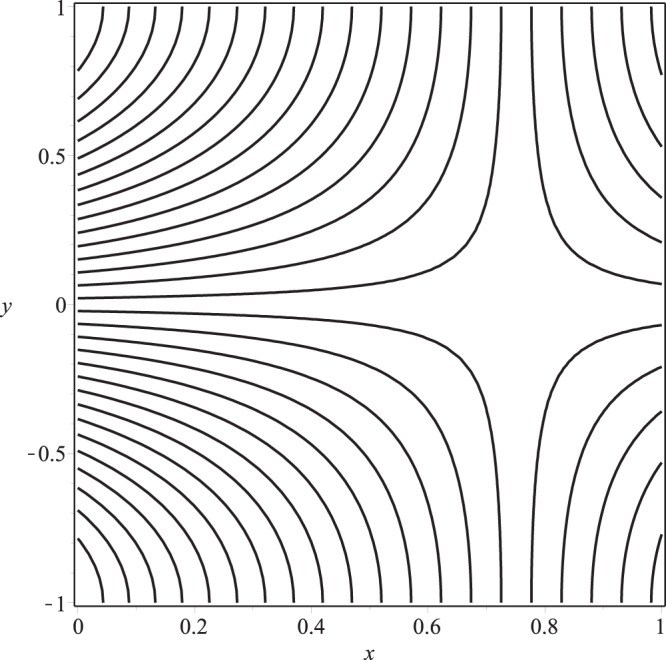
Streamline pattern of the flow for *p*_*i*_ = 3, *K* = 0.25, *λ*_1_ = 0.1, *λ*_2_ = 0.03.

**Figure 16:**
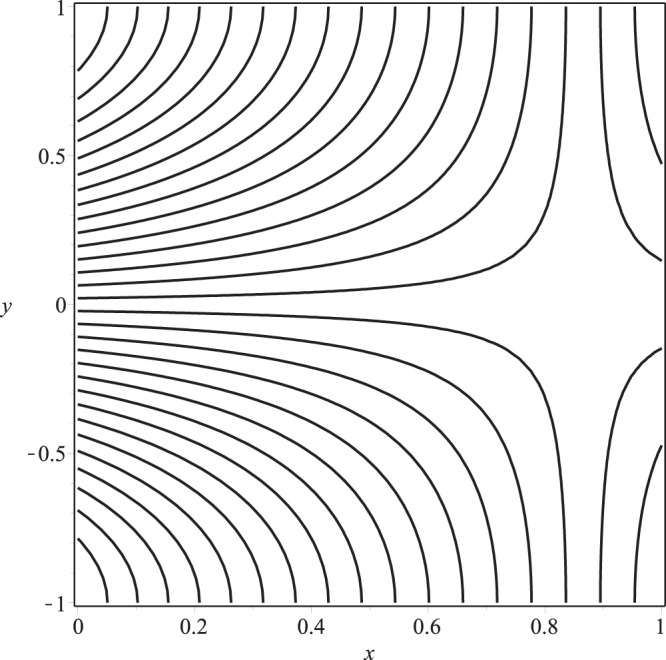
Streamline pattern of the flow for *p*_*i*_ = 4, *K* = 0.16, *λ*_1_ = 0.1, *λ*_2_ = 0.03.

**Figure 17:**
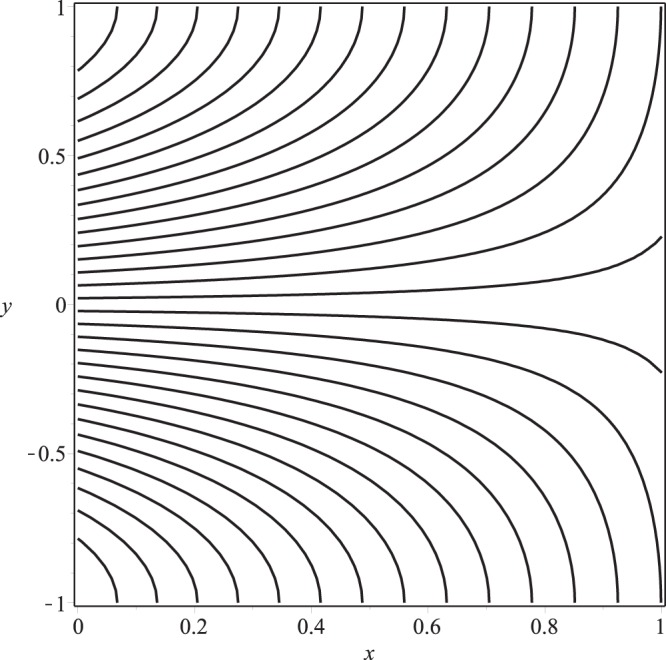
Streamline pattern of the flow for *p*_*i*_ = 3, *K* = 0.16, *λ*_1_ = 2, *λ*_2_ = 0.03.

Streamline pattern of the flow is presented in Figs 14−17. It is observed from the Fig. 14 that the flow is positive axial through the length of channel and no reverse flow and reverse leakage occurs for prescribed values of the parameters. However, as the wall permeability parameter *K* and (or) the inlet pressure *p*_*i*_ increase to a certain value, a reverse flow phenomenon occurs. It happens due to the fact that if *K* or *p*_*i*_ are increased, all the entering fluid is reabsorbed through seepage at the channel wall before the channel exit. Thus, a stagnation point flow can be seen in these two figures, Figs 15 and 16. A comparison of Figs 14 and 17 show that a very nominal increase in the fluid seepage through channel walls happen as the value of *λ*_1_ is increased.

## Application to Flat Plate Hemodialyzer

In this section, we aim to derive equations for the computation of ultrafiltration rate *Q*_*A*_ and the mean pressure drop Δ*P* in an artificial kidney (FPD) by using results of the former section. An FPD comprises of several compartments of blood. Every compartment involves two rectangular sheets that are composed of recovered cellulose. The two sheets are clipped at their edges by a couple of rectangular notched plastic sheets. The blood streams between the cellulose sheets whereas the dializing liquid goes in a counter-current or a cross-current stream along the sections in hemodialyzer board^6,27–29^. The volume of blood lost by the leakage through cellulose in a given time, from a known recycling volume is taken to be the ultrafiltration rate.

Let *L* be the cellulose length, then we can compute the ultrafiltration rate for the presented model as following

65 $${\tilde{Q}}_{A}={\tilde{Q}}_{0}-{\tilde{Q}}_{L},$$

where sign denotes the dimensional quantity, *Q*_0_ = *Q*(0) and *Q*_*L*_ = *Q*(*L*). The non-dimensional expression for ${\tilde{Q}}_{A}$ can be obtained using the dimensionless parameters defined earlier. Making use of (46), (49) and (60) in (65), the following expression for the ultrafiltration rate is determined

66 $$\begin{array}{rcl}{Q}_{A} & = & {Q}_{0}-{Q}_{1},\\  & = & 1-\frac{1}{5}(5-{\lambda }_{2}\,K{p}_{i})\cosh \,\xi -\frac{1}{5}{\lambda }_{2}K{p}_{i}\,\cosh \,2\xi \\  &  & -\frac{\xi }{30A}[3{\lambda }_{2}+(4{A}^{2}K{{p}_{i}}^{2}{\lambda }_{2}-20{A}^{2}{p}_{i})(1+{\lambda }_{1})]\sinh \,\xi \\  &  & +\frac{\xi {\lambda }_{2}}{60A}[3+4{A}^{2}K{{p}_{i}}^{2}(1+{\lambda }_{1})]\sinh \,2\xi .\end{array}$$

A similar approach can be adopted to evaluate the expression for calculation of mean pressure drop in FPD between *x* = 0 and *x* = *L*. By using Eq. (58) the following expression can be obtained for the mean pressure drop in a flat plate hemodialyzer

67 $$\begin{array}{rcl}\Delta p(1) & = & \bar{p}(0)-\bar{p}(1),\\  & = & {p}_{i}+\frac{\xi }{10AK}(5-{\lambda }_{2}\,K{p}_{i})\sinh \,\xi +\frac{\xi {p}_{i}{\lambda }_{2}}{5A}\,\sinh \,2\xi \\  &  & +\frac{1}{20{A}^{2}(1+{\lambda }_{1})}[3{\lambda }_{2}+(4{A}^{2}K{{p}_{i}}^{2}{\lambda }_{2}-20{A}^{2}{p}_{i})(1+{\lambda }_{1})]\cosh \,\xi \\  &  & -\frac{{\lambda }_{2}}{20{A}^{2}(1+{\lambda }_{1})}[3+4{A}^{2}K{{p}_{i}}^{2}(1+{\lambda }_{1})]\cosh \,2\xi .\end{array}$$

For checking the accuracy of these expressions, we are employing the experimental data provided in^5,29^ as shown in Table 1. This data corresponds to a disposable flat-plate artificial kidney and is also referred to as the data for an RP kidney. By substituting these parameters in Eq. (66) along with *λ*_1_ = 0.1 and *λ*_2_ = 0.03^21–23^ results in an equation with one variable *K*. By expanding the hyperbolic functions in this equation in the power series of *K* up to *O*(*K*^3^) and then solving the resulting equation we obtain a real root *K* = 6.23 × 10^−4^. The ultrafiltration coefficient *L*_*p*_ is then computed from $K=\frac{{L}_{p}{L}^{2}}{{a}^{3}t}$. This results in *L*_*p*_ = 6.66 × 10^−16^ cm^2^ (see Table 2).

**Table 2:** Computed values for the proposed Jeffrey fluid model.

Parameter	Abbreviation	Numerical Value
Mechanical filtration coefficient	*L*_*p*_	6.66 × 10^−16^ cm^2^
Dimensionless Mechanical filtration coefficient	*K*	6.23 × 10^−4^
Mean pressure drop	$\bar{p}(0)-\bar{p}(L)$	11.55 mm Hg

In a similar way by substituting *K* = 6.23 × 10^−4^ and the parameters given in Table 1 along with *λ*_1_ = 0.1 and *λ*_2_ = 0.03 in Eq. (67), mean pressure drop in an FPD can be computed. This results in the mean pressure drop $\bar{p}(0)-\bar{p}(L)$ = 11.5 mm Hg (see Table 2).

In the data for membranes of hemodialyzer, filtration coefficient *L*_*p*_ value is usually not given. Results of experiments performed by Kaufmann *et al*.^30^ show that at the normal body temperature regenerated cellulose has the hydraulic permeability equal to 2.41 × 10^−11^ cm^3^/dynes-sec when the membrane has a thickness equal to 7.5 × 10^−3^ cm. Taking the fluid viscosity equal to 6.9 × 10^−3^ dynes-sec/cm^2^ from Table 1, this results in *L*_*p*_ = 1.25 × 10^−15^ cm^2^. The ultrafiltration coefficient computed by the empirical results of Marshall *et al*.^5^ shows that *Lp* = 6.36 × 10^−16^ cm^2^. It is also revealed in the experiments discussed in^5,29^ that the mean pressure drop in an FPD is approximately 15 mm Hg. Thus a good agreement between the presented and earlier obtained experimental and empirical values of the ultrafiltration coefficient and mean pressure drop is found. This builds a confidence in stating that the presented model can be used to obtain theoretical results in advance to study the hydrodynamical aspects of the flow in a flat plate hemodialyzer.

**Figure 18:**
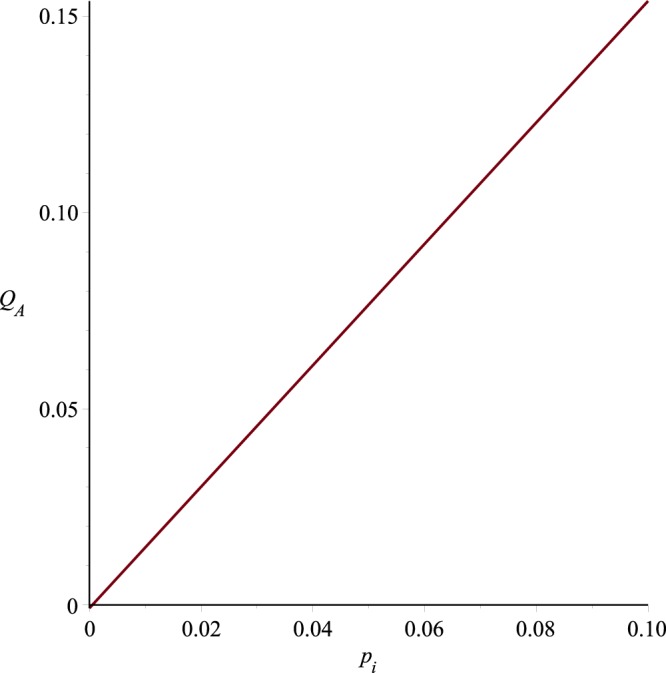
Ultrafiltration rate versus *p*_*i*_, *K* = 0.0006, *A* = 1288.

**Figure 19:**
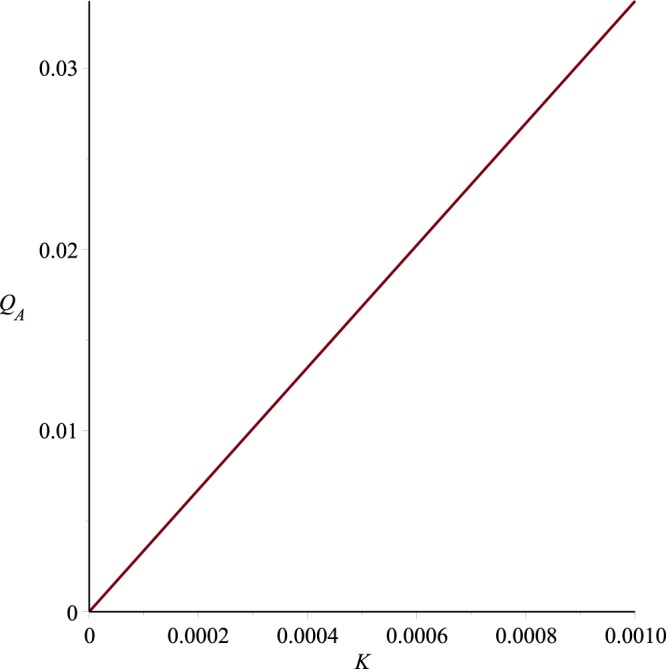
Ultrafiltration rate versus *K*, *p*_*i*_ = 0.013, *A* = 1288.

**Figure 20:**
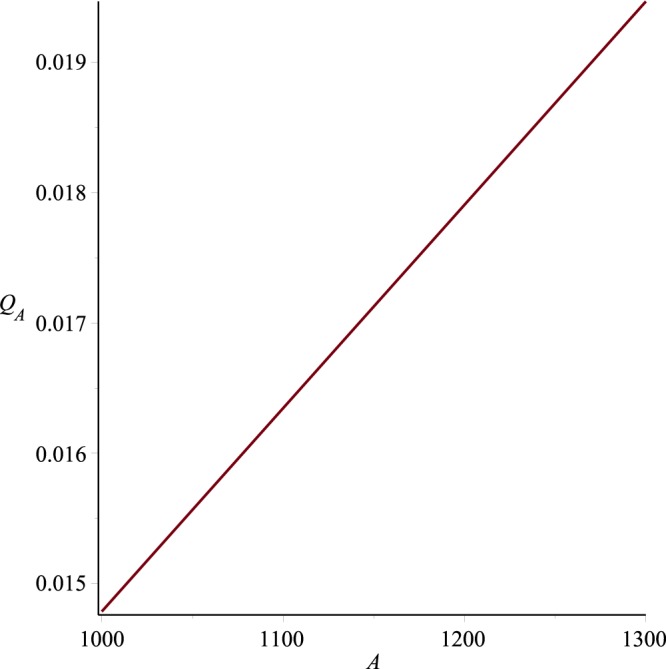
Ultrafiltration rate versus A, *p*_*i*_ = 0.013, *K* = 0.0006.

Graphs of the ultrafiltration rate *Q*_*A*_ versus *p*_*i*_, *K* and *A* are plotted in Figs 18−20, for fixed values of other parameters. These graphs state that *Q*_*A*_ depends linearly on *p*_*i*_, or alternatively the ultrafiltration rate is linearly dependent on the trans-membrane pressure difference $\bar{p}(0)-{p}_{m}$ . It is also evident from these graphs that *Q*_*A*_ is directly proportional to the dimensionless filtration coefficient *K*. Expressions for the dimensionless parameters and Fig. 19 also reveal that the ultrafiltration rate is directly proportional to the mechanical filtration coefficient *L*_*p*_ and membrane area, and is inversely proportional to the membrane thickness and channel half width. Dependence of the ultrafiltration rate on the dimensionless filtration coefficient is also presented in Table 3. The linear dependence of *Q*_*A*_ on *p̄*(0)−*p*_*m*_ have been found experimentally by Malino *et al*.^31^ and Mcdonald^32^. A series of experiments performed by Brown *et al*.^33^ also highlights the dependence of *Q*_*A*_ on the mechanical filtration coefficient, membrane thickness and the membrane area.

**Table 3:** Dimensionless ultrafiltration rate in a flat plate hemodialyzer.

Wall Permeability	0.008	0.010	0.012	0.014	0.016	0.018
Ultrafiltration Rate	0.40	0.50	0.60	0.70	0.80	0.91

**Figure 21:**
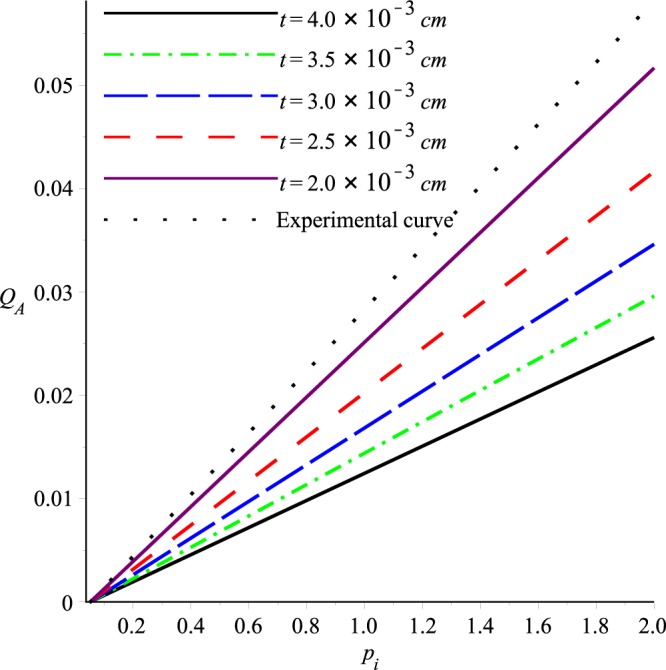
Ultrafiltration rate versus *p*_*i*_ for different membrane thicknesses.

For a given or desired mechanical filtration coefficient *L*_*p*_, Eq. (66) can also be used to determine the magnitude of membrane thickness. This fact is explained in Fig. 21 which shows the behavior of dimensionless ultrafiltration rate *Q*_*A*_ with the trans-membrane pressure difference for different values of membrane thickness. The experimental curve in this graph is plotted by admitting the experimental result of Kauffmann *et al*.^30^ which states that *L*_*p*_ is 1.25 × 10^−15^ cm^2^. From this figure it can be seen that for experimental and theoretical curves to be in good agreement, the membrane thickness *t* should be approximately 1.5.0 × 10^−3^ *cm*. This thickness is approximately half of the estimated thickness obtained from the data of Funck-Bretano *et al*.^29^. The importance of membrane thickness in determining the ultrafiltration rate can also be seen from this graph. For example, it can be seen that magnitude of the ultrafiltration rate can be doubled if the membrane thickness is halved. In order to determine a desired ultrafiltration rate for variations of the dimensionless filtration coefficient *K*, theoretical values of the ultrafiltration rate are tabulated in Table 3. It can be seen that increase in *K* causes the higher ultrafiltration rate.

## Conclusions

The Jeffrey fluid model is a simpler model that describes well the physiological flows of non-Newtonian nature. In the study of fluid flow problems in porous-walled channels of small width, the presented model can serve as generalization of the usual Newtonian fluid model, since the obtained results can be reduced to the later one’s by substituting parameters *λ*_1_ and *λ*_2_ equal to zero. The derived equations for the ultrafiltration rate and the mean pressure difference can be confidently used in studying the flow in a flat plate hemodialyzer. In applying the current results to study the problem of flow in flat plat hemodialyzer, one should not overlook the physical aspects of the flow phenomenon. It is also concluded the presented results are theoretical in their soul, therefore one should perform more experimental and theoretical investigation in order to have a complete understanding of the flow in a flat plat hemodialyzer.
